# Association Between Follistatin and PAI-1 Levels in MASLD Subjects Undergoing a Plant-Based Dietary Intervention

**DOI:** 10.3390/nu17132124

**Published:** 2025-06-26

**Authors:** Nicole Cerabino, Caterina Bonfiglio, Martina Di Chito, Rosanna Donvito, Francesco Pio Mongelli, Pasqua Letizia Pesole, Dolores Stabile, Endrit Shahini, Marianna Zappimbulso, Raffaele Cozzolongo, Gianluigi Giannelli, Giovanni De Pergola

**Affiliations:** 1 Center of Nutrition for the Research and the Care of Obesity and Metabolic Diseases, National Institute of Gastroenterology IRCCS “Saverio de Bellis”, Castellana Grotte, 70013 Bari, Italy; martina.dichito@irccsdebellis.it (M.D.C.); rosanna.donvito@irccsdebellis.it (R.D.); francescomongio@gmail.com (F.P.M.); giovanni.depergola@irccsdebellis.it (G.D.P.); 2 Unit of Data Science, National Institute of Gastroenterology “Saverio de Bellis”, IRCCS Hospital, Castellana Grotte, 70013 Bari, Italy; 3 Core Facility Biobank, National Institute of Gastroenterology “Saverio de Bellis”, IRCCS Hospital, Castellana Grotte, 70013 Bari, Italy; letizia.pesole@irccsdebellis.it (P.L.P.); dolores.stabile@irccsdebellis.it (D.S.); 4 Department of Gastroenterology, National Institute of Gastroenterology “Saverio de Bellis”, IRCCS Hospital, Castellana Grotte, 70013 Bari, Italy; endrit.shahini@irccsdebellis.it (E.S.); marianna.zappimbulso@irccsdebellis.it (M.Z.); raffaele.cozzolongo@irccsdebellis.it (R.C.); 5 Scientific Direction, National Institute of Gastroenterology “Saverio de Bellis”, IRCCS Hospital, Castellana Grotte, 70013 Bari, Italy; gianluigi.giannelli@irccsdebellis.it

**Keywords:** follistatin, PAI-1, MASLD, diet, vegetables

## Abstract

**Background**: Metabolic dysfunction-associated steatotic liver disease (MASLD) is a chronic liver condition intricately linked to systemic metabolic impairments. Among the molecular mediators implicated in its pathogenesis, follistatin and plasminogen activator inhibitor-1 (PAI-1) play a significant role in inflammatory, fibrotic, and metabolic processes. However, the interplay between these two biomarkers in the context of MASLD remains poorly understood. **Objective**: This study analyzes the relationship between follistatin and PAI-1 in subjects with MASLD and obesity. It also assesses changes in these biomarkers and metabolic parameters after a dietary intervention that involves increasing one serving of vegetables and reducing one serving of carbohydrates. **Methods**: Forty-four individuals with MASLD and obesity participated in a two-month dietary intervention. The concentrations of PAI-1 and follistatin were measured at baseline and post-intervention. Multivariate linear regression models, adjusted for age, gender, waist circumference, and insulin resistance (measured by HOMA-IR), were employed to analyze the association between the two biomarkers. **Results**: Following the dietary intervention, PAI-1 levels showed a significant reduction (from 35.76 to 33.54 ng/mL; *p* < 0.001), whereas follistatin concentrations remained relatively stable (from 43.6 to 45.3 ng/mL; *p* = 0.392). Post-intervention, multivariate analysis reveals that higher follistatin levels were independently associated with lower PAI-1 levels. The inclusion of follistatin in the regression model enhanced the estimated dietary effect on PAI-1 reduction (from –0.145 to –0.194), suggesting a possible independent modulatory role of follistatin in the regulation of PAI-1 levels. **Conclusions**: These findings indicate that follistatin may act as an inhibitory regulator of PAI-1 expression in individuals with MASLD and obesity, potentially contributing to reductions in the prothrombotic status during dietary intervention. The data suggest a synergistic relationship between follistatin and PAI-1 in the regulation of prothrombotic status in conditions of hepatic steatosis.

## 1. Introduction

Metabolic dysfunction-associated steatotic liver disease (MASLD) has emerged as one of the most prevalent forms of chronic liver disease globally. This updated nomenclature replaced the previous term “non-alcoholic fatty liver disease” (NAFLD) to underscore the central role of systemic metabolic disturbances—such as visceral adiposity, insulin resistance, and dyslipidemia—in the etiology of hepatic steatosis [1]. Rather than a static diagnosis, MASLD encompasses a progressive clinical spectrum that may evolve into metabolic steatohepatitis (MASH), fibrosis, cirrhosis, and hepatocellular carcinoma [2].

The rising global burden of MASLD parallels the increasing prevalence of obesity, metabolic syndrome, and type 2 diabetes mellitus [2]. Pathophysiologically, the condition is characterized by intrahepatic lipid accumulation, alongside a cascade of events, including insulin resistance, lipotoxicity, oxidative stress, activation of pro-inflammatory and fibrogenic pathways, and dysregulation of both innate and adaptive immunity [3]. Progression from simple steatosis to more advanced stages of liver disease involves complex interactions between systemic metabolic processes and localized hepatic responses.

Identifying circulating biomarkers that reflect these metabolic, inflammatory, and fibrogenic reactions is essential to elucidate the MASLD pathogenesis and develop precision-based therapeutic strategies. Among the biomarkers of interest, plasminogen activator inhibitor-1 (PAI-1) has been widely studied for its dual role in inhibiting fibrinolysis and promoting fibrotic and inflammatory responses in metabolic contexts [4]. Produced by hepatocytes, adipocytes, and endothelial cells, PAI-1 is upregulated by pro-inflammatory cytokines such as TNF-α, IL-1β, and TGF-β, which are commonly elevated in MASLD [5]. Elevated PAI-1 levels are associated with insulin resistance, endothelial dysfunction, liver fibrosis, and elevated cardiovascular risk, making this inhibitor a key systemic indicator of metabolic derangement [6]. Very recently, PAI-1 production and blood levels have been suggested to be increased in contexts of liver steatosis [7].

Follistatin, a glycoprotein member of the transforming growth factor-beta (TGF-β) superfamily, acts by binding and inhibiting activins and myostatin, thereby modulating diverse biological processes, including inflammation, cellular proliferation, and energy metabolism [8].

Activins, particularly activin A, are involved in immune modulation, fibrosis, and metabolic regulation, often exerting pro-inflammatory and insulin-desensitizing effects.

Myostatin is a key inhibitor of skeletal muscle growth and is also involved in systemic energy regulation; elevated myostatin levels are associated with reduced muscle mass, increased adiposity, and impaired insulin sensitivity [9,10]. By antagonizing these factors, follistatin exerts broad anabolic and anti-inflammatory effects.

While initially characterized in the context of muscle homeostasis, emerging evidence suggests that follistatin plays an active role in hepatic and systemic metabolic regulation, influencing insulin sensitivity, body composition, and energy balance [11,12].

Specifically, follistatin contributes to energy homeostasis by promoting skeletal muscle hypertrophy, which increases the basal metabolic rate and glucose uptake. It also enhances lipid utilization through the stimulation of fatty acid oxidation in the liver and skeletal muscle. Furthermore, follistatin promotes the browning of white adipose tissue, leading to increased thermogenesis and energy expenditure [11]. These combined effects suggest that follistatin acts not only as a regulator of muscle mass but also as a metabolic modulator, improving systemic energy efficiency and flexibility under conditions of metabolic stress or insulin resistance.

Its expression is regulated by key metabolic hormones, such as insulin, glucagon, and pro-inflammatory cytokines, and elevated circulating levels are frequently observed in individuals with obesity, type 2 diabetes, and other metabolic disorders, potentially reflecting a compensatory response aimed at mitigating an energy imbalance and insulin resistance [13].

A biologically plausible link exists between follistatin and PAI-1 in MASLD, as both are modulated by overlapping inflammatory and metabolic pathways and may converge in regulating fibrosis, inflammation, and tissue remodeling. Their expression is influenced by systemic inflammatory stimuli commonly present in MASLD patients [14,15], and modulation of the activin–follistatin axis has been shown to affect TGF-β1 signaling, a well-known inducer of PAI-1 expression and a key driver of hepatic fibrogenesis [16].

Notably, in vitro studies have demonstrated that activins enhance PAI-1 production, an effect that is mitigated by follistatin through inhibition of receptor–ligand interactions [17]. This suggests that follistatin may act as a negative regulator of PAI-1 synthesis, potentially influencing the fibrotic milieu of the liver.

Importantly, both PAI-1 and follistatin are responsive to environmental changes, such as diet and physical activity, highlighting their potential as dynamic biomarkers. These features make them valuable both for understanding MASLD pathophysiology and monitoring responses to lifestyle-based interventions.

Accordingly, the present study investigates the association between follistatin and PAI-1 levels in individuals with MASLD and obesity undergoing a controlled two-month dietary intervention. The aim is to evaluate whether follistatin serves as an independent modulator of PAI-1 expression and explore whether changes in PAI-1 levels may be partially mediated by alterations in follistatin concentrations. By exploring this potential molecular interplay, the study seeks to contribute to the understanding of metabolic–inflammatory regulation in MASLD and inform future nutritional strategies for disease management.

## 2. Materials and Methods

### 2.1. Study Design and Population

This prospective two-month study was carried out by the Clinical Nutrition Research Center for Obesity and Metabolic Diseases at the National Institute of Gastroenterology “Saverio de Bellis” (Castellana Grotte, Bari, Italy). Eligible participants were aged 18 to 65 years, with a BMI exceeding 30 kg/m^2^, who were not taking any current medications. Exclusion criteria included diagnosed or newly detected diabetes mellitus, cardiovascular diseases, respiratory failure, severe gastrointestinal conditions, chronic kidney disease (e.g., estimated glomerular filtration rate < 60 mL/min/1.73 m^2^), psychiatric disorders, pregnancy or lactation, eating disorders, chronic liver diseases of non-metabolic origin, alcohol intake exceeding 30 g/day for men or 20 g/day for women, substance abuse, infectious diseases, acute illnesses affecting inflammatory markers, rare metabolic diseases, or mitochondrial fatty acid oxidation disorders.

Participants categorized as obese underwent anthropometric and bioimpedance evaluations, clinical history assessments, and laboratory analyses (hematological and biochemical parameters). Physical activity levels were evaluated using the International Physical Activity Questionnaire (IPAQ) [18]. Dietary adherence to the Mediterranean diet was assessed using the PREDIMED questionnaire [19]. Smoking habits were also recorded.

The study protocol was approved by the local Medical Ethics Committee (Prot. no. 681 of 15 November 2023) and conducted in accordance with the ethical standards set by the 1964 Declaration of Helsinki. All participants provided written informed consent before enrollment. Recruitment took place between May 2023 and December 2024. Two clinical visits were conducted: baseline (T0) and after two months (T1). During both visits, data collection included fasting blood samples, anthropometric assessments, and instrumental evaluations (BIA, InBody Co., Ltd., Seoul, Republic of Korea, and FibroScan, Echosens, Paris, France).

Participants were instructed to complete a three-day food diary, including two weekdays and one weekend day. After assessing usual dietary intake, participants were asked to eliminate one daily portion of carbohydrates (e.g., bread, pasta, or potatoes) to be replaced by a 200 g serving of vegetables. No other lifestyle or dietary changes were recommended. The vegetables included four *Brassicaceae* species: *Brassica rapa* var. *cymosa*, *Cichorium intybus*, *Brassica oleracea* L. var. *sabellica*, and *Sinapis arvensis* var. *orientalis* [20]. These vegetables are known to be rich in bioactive compounds such as isothiocyanates and phenolic substances, which have demonstrated antioxidant and anti-lipogenic properties in preclinical studies on NAFLD [21].

### 2.2. MASLD Assessment

FibroScan is a reliable, cost-effective, and non-invasive method for assessing hepatic steatosis and fibrosis in at-risk individuals [22]. Although liver biopsy remains the diagnostic gold standard for evaluating steatosis, fibrosis, and inflammation, ultrasound-based elastography with FibroScan provides a painless and comprehensive liver assessment and is recommended as a first-line diagnostic tool [23].

Vibration-controlled transient elastography (VCTE) combined with a Controlled Attenuation Parameter (CAP) at 3.5 MHz was used to estimate the hepatic fat content. CAP thresholds for grading steatosis were 248 dB/m for mild, 268 dB/m for moderate, and 280 dB/m for severe steatosis [24]. Liver stiffness measurement (LSM) was used to evaluate liver fibrosis, with thresholds of 8 kPa and 12 kPa indicating fibrosis and advanced fibrosis (stage 3), respectively.

### 2.3. Anthropometric Parameters

Height and weight were measured in fasting participants wearing light clothing, who were barefoot and had an empty bladder. BMI was calculated using standardized equipment for all participants. Waist circumference was measured at the midpoint between the lower costal margin and the iliac crest. Systolic and diastolic blood pressure (SBP and DBP) were measured three times with patients seated at rest using an automatic blood pressure monitor (OMRON M6, Kyoto, Japan).

### 2.4. Bioelectrical Impedance Analysis (BIA)

Bioelectrical impedance was assessed using a single-frequency analyzer (BIA-101, 50 kHz; Akern Bioresearch, Florence, Italy). Following ESPEN guidelines [25], participants were examined in the supine position, with their legs slightly apart. They were asked to refrain from eating, exercising, or consuming alcohol for at least 12 h before the test. After cleaning the skin with alcohol, injector electrodes were placed on the dorsal surface of the right hand and the superior surface of the right foot, while sensor electrodes were placed on the distal right wrist and between the medial and lateral malleoli of the right ankle [26]. Resistance (RZ) and reactance (Xc) were measured, and body composition parameters—fat mass (FM), fat-free mass (FFM), total body water (TBW), and extracellular water (ECW)—were calculated using specific software algorithms.

### 2.5. Biochemical Analyses

Fasting blood samples were drawn between 8:00 and 9:00 a.m. Serum samples were analyzed for fasting serum glucose (FSG), fasting insulin, triglycerides, total cholesterol, LDL-C, HDL-C, AST, ALT, GGT, uric acid, creatinine, high-sensitivity *C*-reactive protein, thyroid function parameters, and 25-hydroxyvitamin D. Analyses were performed using the COBAS 8000 autoanalyzer (ROCHE Diagnostic SPA, Monza, Italy).

Glycated hemoglobin (HbA1c) was measured with the Capillarys 3 OCTA capillary electrophoresis system (Sebia Italia S.r.l., Bagno a Ripoli, Florence, Italy).

Insulin resistance was estimated using the Homeostasis Model Assessment of Insulin Resistance (HOMA-IR), calculated by the following formula [27]:

HOMA-IR = FSG (mg/dL) × fasting insulin (μIU/mL)405.

PAI-1 serum levels were determined by ELISA assay, according to the manufacturer’s instructions (Human PAI-1 ELISA kit, Invitrogen, Vienna, Austria). The analytical sensitivity of the assay is <30 pg/mL human PAI-1.

Follistatin serum levels were determined by ELISA assay, according to the manufacturer’s instructions (Human Follistatin ELISA kit, Invitrogen, Vienna, Austria). The minimum detectable dose of follistatin is 500 pg/mL.

### 2.6. Variables of Exposure and Confounders

The exposure variable was a plant-enriched diet intervention. Five confounding parameters, gender, age (<50 vs. ≥50 years), follistatin, HOMA-IR (<2.5 vs. ≥2.5), and waist circumference, were considered in the final multivariate statistical model to adjust the association estimates between the effects of the dietary intervention in the two months of treatment and PAI1.

### 2.7. Statistical Methods

We performed statistical analysis of baseline variables, expressed as mean ± Standard Deviation (SD), median, and range for continuous variables. We used the Wilcoxon rank sum test, by paired data, for continuous variables to compare two groups and the χ^2^ test to evaluate differences for categorical variables. Statistical significance was determined by 95% Confidence Intervals (CIs) for *p*-values of 0.05 or less. Additionally, a retrospective power analysis was conducted to demonstrate that our sample was adequate and, therefore, the results were reliable.

The correlation coefficient, with relative *p*-values, between the differences before and after the dietary intervention for PAI 1 and follistatin was calculated and plotted in the linear prediction plot.

The correlation coefficient is a statistical measure that quantifies the strength and direction of the relationship between two variables. Specifically, it indicates whether the two variables are positively correlated (when one increases, the other increases), negatively correlated (when one increases, the other decreases), or if there is no correlation [28].

We also employed a scatter plot alongside the linear graph to represent the values of ∆PAI1 and ∆Follistatin. The position of each point on the horizontal and vertical axes indicates the values of a single data point. A scatter plot utilizes points to depict the values of two different numerical variables.

A Generalized Estimating Equation (GEE) [29] was used to estimate the longitudinal trajectories of PAI1 (pre- and post-plant-enriched diet).

GEE models help estimate mean changes in biomarker values while adjusting for covariates in biomedical investigations because they allow correlations of response data (repeated measurements in each subject). Due to the non-normal distribution of the outcome variables, a gamma distribution (link identity) was used to model the response, and an unstructured correlation matrix was applied to the data.

Initially, confounding variables were selected from the existing literature. Then, the minimum absolute reduction and selection (LASSO) was adopted to reduce the number of candidate predictors and select those most useful for the model construction [30].

The Time Diet Intervention was considered the exposure variable, while gender, age (<50 vs. ≥50 years), follistatin, HOMA (<2.5 vs. ≥2.5), and waist were all considered as covariates.

Three models were constructed to estimate changes in the effect of diet on PAI1: model a was univariate, model b was adjusted for gender and age, and model c was adjusted for follistatin, gender, age (<50 vs. ≥50 years), HOMA (<2.5 vs. ≥2.5), and waist circumference.

Stata statistical software version 19.0 (StataCorp 2025, 4905 Lakeway Drive, College Station, TX 77845, USA) was used for statistical analysis.

## 3. Results

In our sample, there were equal numbers of men and women. The mean age of women was 45.15 years (11.46) versus 47.71 (9.49) for men. All subjects were classified as a continuum of obese subjects. Women had a mean BMI at baseline of 36.6 (4.88), which reduced to 34.74 (4.55) at the end of two months, while men had a BMI at baseline of 36.79 (3.65) reduced to 34.79 (3.78) at the end of the dietary intervention.

Table 1 shows the baseline demographic and lifestyle characteristics, while Table 2 displays parameters for the whole sample pre- and post-diet.

Table 2 shows the entire sample data before and after the plant-enriched diet. CAP (the FibroScan parameter of steatosis) and liver stiffness (the FibroScan parameter of fibrosis) in the liver were lower after the diet. At the end of the dietary intervention period, an overall improvement in all measured values was observed, especially for systolic and diastolic blood pressure, BMI, waist circumference, fat mass, insulin, HOMA, glycated hemoglobin, triglycerides, total and LDL cholesterol, ALT, AST, and γGT, which were all significantly reduced.

**Figure 1:**
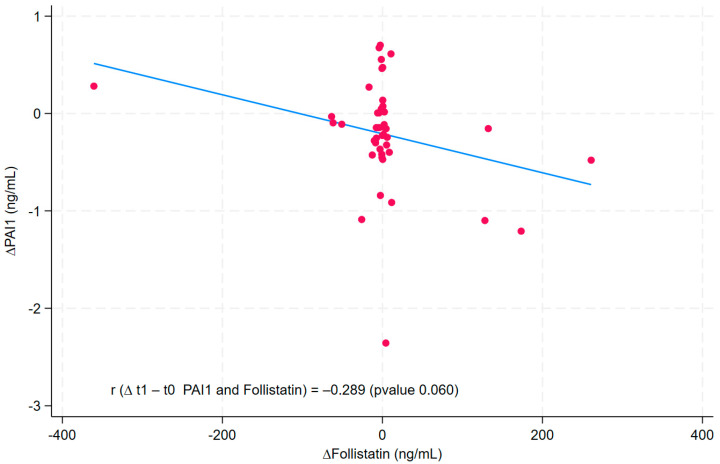
Linear and scatter plot between ∆PAI1 and ∆Leptin. r: correlation coefficient; PAI1: plasminogen activator inhibitor-1. The scatter plot represents the relationship between ∆PAI1 and ∆Follistatin. The Cartesian coordinates (red dots) represent the values of the two variables for a single observation. The blue line identifies the trend of the dots, the type and strength of the correlation between the two variables.

The linear plot (Figure 1) shows the inversely proportional relationship between ∆PAI1 (PAI1 post-diet–PAI1 pre-diet) and ∆Follistatin (follistatin post-diet–follistatin pre-diet), with a correlation coefficient (r) of −0.288 (*p*-value: 0.061). We also used a scatter plot to represent the values of ∆PAI1 and ∆Follistatin. The position of each point on the horizontal and vertical axis indicates the values of a single data point.

Table 3 shows the results of the three regression analysis models built with a GEE to illustrate the associations between the effect of a plant-enriched diet and PAI1 in patients with MASLD. A statistically significant association of the effect of diet was observed in model a (β = −0.145; *p*-value = 0.041). As model *a* is univariate, the beta regression coefficient, which estimates the mean decrease in PAI before and after follow-up, is based solely on the effect of the diet, neglecting all potential confounding variables.

When correcting for gender and age, as in model b, the decrease in PAI1 before and after the diet remains almost constant, as indicated by the value of the coefficient: β = −0.146 (*p*-value = 0.040). Therefore, age and gender do not contribute to the decrease in PAI1 before and after the diet.

Upon analyzing the multivariate model *c*, the estimated effect of the diet on PAI1, represented by the beta coefficient, was −0.194, with a *p*-value of 0.028, while the effect of follistatin on PAI1 was β = −0.002 (*p*-value: 0.002). In other words, before and after the plant-based diet, there was a statistically significant reduction in PAI1 of 0.194 ng/mL, and for each unit increase in follistatin, there was a reduction in PAI1 of 0.002 ng/mL, controlling for gender (female vs. male), age (<50 vs. ≥50 years), waist circumference, and HOMA (<2.5 vs. ≥2.5).

Thus, there is a greater reduction in PAI1 at the end of dietary treatment compared with the previous regression models *a* and *b*, attributable to the inclusion in model *c* of HOMA, waist circumference, gender, and age but mainly due to follistatin (see Table 3).

## 4. Discussion

This study demonstrates that a two-month dietary intervention based on supplementation with specific plants belonging to the *Brassicaceae* family resulted in a clinically meaningful reduction in serum PAI-1 levels in subjects with MASLD. In the simple pre-post comparison, a statistically significant reduction (from 35.76 to 33.54 ng/mL *p* < 0.001) of PAI-1 was observed; multivariate analysis with GEE models further highlights this significant reduction associated with dietary intervention. In particular, the estimated effect of the intervention increased after the inclusion of follistatin in the model, showing a decrease of −0.194 ng/mL (*p* = 0.028) in subjects who followed the diet, suggesting a potential role of the latter as a modulator of PAI-1 expression. In addition, the identification of an inverse association between follistatin and PAI-1 levels suggests an independent regulatory mechanism by which follistatin might modulate hepatic PAI-1 expression or secretion.

### 4.1. Follistatin as a Modulator of Inflammation and Fibrosis

Follistatin, mainly known for its activin-binding and activin-inhibiting function, has gained increasing interest in the hepatological field due to its involvement in inflammatory, fibrotic, and metabolic processes. The activin–follistatin axis is indeed recognized as a critical node in the regulation of the inflammatory response and the progression of liver fibrosis through modulation of the TGF-β1 [8] signaling pathway. Activins stimulate PAI-1 transcription through type II receptors and the Smad2/3 [16] cascade; follistatin, by competitively binding to activins, prevents this activation, thereby reducing PAI-1 expression. Our results, showing a negative association between follistatin and PAI-1, are consistent with this model and suggest the indirect action of follistatin in reducing the pro-fibrotic environment of the liver under conditions of metabolic dysfunction.

Although plausible pathways have been hypothesized, such as follistatin-mediated TGF-β1 inhibition, future mechanistic studies and in vitro validations will be needed to confirm and elucidate these biological hypotheses.

It is noteworthy that the absolute levels of follistatin did not change significantly after the intervention (*p* = 0.392). However, its inclusion in the statistical model significantly improved the effect of diet on PAI-1, reinforcing the hypothesis that follistatin acts as an endogenous modulator affecting the biological response to nutritional intervention.

Methodologically, the inclusion of confounding variables, such as waist circumference, HOMA-IR, and follistatin, strengthened the internal validity of the results.

### 4.2. PAI-1 as a Metabolic and Inflammatory Target

The reduction in PAI-1 is particularly relevant considering its role as a central mediator in chronic inflammation, fibrosis, and endothelial dysfunction. Elevated PAI-1 levels are associated with increased cardiovascular risk, insulin resistance, and worsening liver fibrosis in patients with metabolic liver steatosis [5,6]. The ability to reduce PAI-1 through relatively simple dietary modifications, therefore, offers a promising strategy for the integrated management of MASLD in the absence of approved drug therapies.

The literature has shown that high levels of PAI-1 are associated with increased vascular stiffness and the progression of liver damage. Therefore, its decrease, even early, could be a favorable indicator. It is plausible that prolonged dietary interventions combined with physical activity could produce even more pronounced and sustainable effects [31].

The improvements observed in metabolic and anthropometric parameters—such as reductions in systolic and diastolic blood pressure, BMI, waist circumference, fat mass, insulin, HOMA, glycated hemoglobin, triglycerides, total and LDL cholesterol, ALT, AST, and γGT—further reinforce the efficacy of the intervention. The significant decrease in the CAP value, from 313.8 to 278.5 dB/m (*p* < 0.001), confirms the improvement in hepatic steatosis, consistent with previous studies that have highlighted the beneficial effects of Mediterranean and plant-based diets on liver composition [20].

Studies with dietary interventions of longer duration (6 months) [31,32,33] have documented similar reductions in inflammatory markers and hepatic steatosis as early as 3 months after intervention. The fact that similar results were also obtained in the present study within a time frame of only eight weeks reinforces the hypothesis that targeted dietary modifications can have rapid and significant effects on the pathophysiology of MASLD.

### 4.3. Role of Diet and Bioactive Compounds

The observed effect on the entire metabolic profile can be attributed, at least in part, to the biochemical composition of the vegetables used, which are known to be rich in isothiocyanates, flavonoids, glucosinolates, and fiber. In particular, sulforaphane, an isothiocyanate derived from glucoraphanin found in cabbage and broccoli, has been shown to have hepatoprotective, antioxidant, and anti-inflammatory effects in animal models of NAFLD and MASLD [34]. The increase in the Mediterranean diet adherence score observed in the participants (from 8 to 11) suggests that the dietary change was well accepted and potentially sustainable in the long term.

### 4.4. Clinical Implications

The possibility of reducing PAI-1 levels through a simple and well-tolerated dietary intervention without the need for pharmacological treatment is particularly relevant in the context of MASLD. Our results indicate that even a small change in dietary quality, such as replacing a portion of refined carbohydrates with vegetables rich in phytocompounds, can have a significant impact on the systemic and local pathophysiology of the disease.

Furthermore, the association between follistatin and treatment response lays the foundation for future research to evaluate its potential as a biomarker of therapeutic response or even as a pharmacological target. The possibility of modulating this protein through nutrition is still an under-explored frontier of precision medicine and nutrigenomics applied to liver disease.

### 4.5. Limitations of the Study

Despite its methodological quality, this study has some limitations. Firstly, the relatively short duration of the intervention may have underestimated slower metabolic effects, such as impacts on the glycemic profile and liver fibrosis. Future studies with extended follow-up will be needed to evaluate the sustainability and clinical relevance of the observed changes. Secondly, the relatively small sample size may have reduced the statistical power.

In addition, we recognize that the absence of a control group represents a limitation of the study design. However, this choice was approved by the ethics committee, considering the nature of the treatment and its feasibility in the clinical setting.

An additional limitation of the study is the absence of a detailed phytochemical analysis of the vegetables consumed and the possible inter-individual variability in their intake. This heterogeneity may have partly influenced the observed effect, making it more difficult to attribute the benefit to a specific bioactive compound. Future studies will need to include the precise quantification of ingested phytocompounds to better define the observed associations.

In view of the good results obtained with this dietary model, it would be interesting to evaluate, in future studies, the effect of other dietary patterns on the same molecules studied.

## 5. Conclusions

The results of this study suggest that a simple but targeted dietary intervention based on increased daily consumption of vegetables belonging to the *Brassicaceae* family may lead to favorable effects in the context of metabolic dysfunction-associated steatotic liver disease (MASLD). These results are in line with previous studies that emphasized the protective value of diets rich in antioxidant phytochemical compounds, such as isothiocyanates and polyphenols contained in *Brassicaceae*, in the context of non-alcoholic hepatic steatosis.

The data obtained make a relevant contribution to understanding the role of dynamic biomarkers such as PAI-1 and follistatin in MASLD and indicate how targeted nutritional approaches can be effective, sustainable, and side-effect-free tools in the early treatment of the disease. In light of the current absence of approved specific drug therapies for MASLD, nutrition emerges as a strategic first-line intervention with the potential to slow disease progression and reduce the risk associated with its metabolic and cardiovascular complications.

Future large-scale, randomized controlled studies using a longer duration of the intervention are desirable in order to investigate the molecular mechanisms underlying the interaction between diet, follistatin, and PAI-1. Such research could provide the basis for the development of personalized therapeutic strategies that include, alongside functional nutrition, possible pharmacological treatments aimed at selectively modulating these molecular axes.

## Figures and Tables

**Table 1:** Demographic and lifestyle characteristics at baseline.

Parameter	Value
N	44
Age * (years)	46.5 (10.4)
Gender (%)	
Female	22 (50)
Male	22 (50)
Smoking habit (%)	
Never	39 (95)
Current	2 (5)
Physical activity (%)	
<30 min	8 (20)
>30 min	26 (63)
Sports person	7 (17)
Education (%)	
Secondary School	11 (27%)
High School	22 (54%)
Graduate	8 (20%)
Snoring (%)	
No	10 (24%)
Yes	31 (76%)
GERD symptoms (%)	
No	25 (61%)
Yes	16 (39%)
Sleepiness (%)	
No	26 (63%)
Yes	15 (37%)

* Mean (SD). GERD: gastro-esophageal reflux disease.

**Table 2:** Description of the whole sample pre- and post-plant-enriched diet.

Parameters	Pre-Diet	Post-Diet	*p*-Value ^¥^
N	44	44	
	Mean (SD)	Mean (SD)	
**Outcome variable:**			
PAI1 (ng/mL)	3.58 (0.90)	3.35 (0.80)	<0.001
**Molecule**			
Follistatin (ng/mL)	43.6 (108.7)	45.3 (80.5)	0.392
**Ultrasonographic measures of liver steatosis and fibrosis**			
FibroScan CAP (dB/m)	313.8 (47.0)	278.5 (48.9)	<0.001
FibroScan LSM (kPa)	6.6 (3.0)	6.4 (4.1)	0.064
**Anthropometric and clinical parameters**			
SBP (mmHg)	133.1 (13.0)	124.5 (8.6)	0.001
DBP (mmHg)	80.6 (11.4)	76.2 (8.1)	<0.001
PREDIMED questionnaire	8.0 (7.0, 9.0)	11.0 (10.0, 12.0)	<0.001
BMI (kg/m^2^)	36.7 (4.2)	34.8 (4.1)	<0.001
Waist circumference (cm)	113.7 (11.9)	107.4 (12.5)	<0.001
Fat mass (kg)	40.9 (10.2)	36.0 (9.4)	<0.001
Free-fat mass (kg)	63.5 (11.8)	62.8 (11.6)	0.125
Body cell mass	35.8 (7.8)	35.6 (7.9)	0.338
**Blood tests:**			
Glucose (mg/dL)	94.5 (8.9)	94.8 (8.4)	0.799
Insulin (µIU/mL)	19.4 (10.4)	15.9 (8.4)	<0.001
HOMA-IR	4.6 (2.7)	3.8 (2.1)	<0.001
Hemoglobin A1C	5.5 (0.4)	5.4 (0.3)	<0.001
Triglycerides (mg/dL)	123.3 (59.5)	101.5 (54.2)	<0.001
Total cholesterol (mg/dL)	192.4 (31.4)	177.3 (29.5)	<0.001
HDL cholesterol (mg/dL)	50.0 (11.4)	47.2 (10.9)	0.008
LDL cholesterol (mg/dL)	125.8 (28.5)	110.9 (25.2)	<0.001
AST (U/L)	22.2 (10.7)	19.2 (7.7)	<0.001
ALT (U/L)	29.9 (18.1)	22.6 (12.5)	<0.001
γGT (U/L)	24.6 (14.4)	20.2 (12.1)	<0.001
Uric acid (mg/dL)	5.4 (1.7)	5.4 (1.3)	0.543
Creatinine (mg/dL)	0.9 (0.2)	0.8 (0.2)	0.592
hs-CRP (mg/dL)	0.3 (0.2)	0.4 (0.7)	0.210
25-hydroxyvitamin D	25.2 (8.0)	22.2 (5.6)	0.003
TSH (µmU/mL)	1.9 (0.9)	1.9 (1.2)	0.083
FT3 (pg/mL)	3.4 (0.5)	3.1 (0.3)	0.002
FT4 (ng/dL)	12.2 (1.2)	11.8 (2.0)	0.423

^¥^ Wilcoxon signed-rank test by paired data. Legend: PAI1: plasminogen activator inhibitor-1; CAP: controlled attenuation parameter; LSM: liver stiffness measurement; SBP: systolic blood pressure; DBP: diastolic blood pressure; PREDIMED questionnaire; BMI: body mass index; HOMA-IR: homeostasis model assessment for insulin resistance; HDL: high-density lipoprotein; LDL: low-density lipoprotein; AST: aspartate aminotransferase; ALT: alanine transaminase; γGT: gamma-glutamyl transpeptidase; hs-CRP: high-sensitivity *C*-reactive protein; TSH: thyroid-stimulating hormone; FT4: free tetraiodothyronine; FT3: free triiodothyronine.

**Table 3:** Generalized estimating equation (GEE): expected values of PAI1 by time (pre- and post-plant-enriched diet).

PAI1 (ng/mL)	β	*p*-Value	95% CI
Model *a*:			
Pre-Diet	0.00		
Post-Diet	−0.145	0.041	−0.285; −0.005
Model *b*:			
Pre-Diet	0.00		
Post-Diet	−0.146	0.040	−0.285; −0.007
Model *c*:			
Pre-Diet	0.00		
Post-Diet	−0.194	0.028	−0.368; −0.021
Follistatin	−0.002	0.002	−0.003; −0.000

Model *a*: univariate. Model *b*: adj for gender (female vs. male) and age (<50 vs. ≥50 years), Model *c*: adj for gender (female vs. male), age (<50 vs. ≥50 years), follistatin, HOMA (<2.5 vs. ≥2.5), and waist circumference. PAI1: plasminogen activator inhibitor-1; β: regression coefficient; CI: confidence interval.

## Data Availability

The original contributions presented in this study are included in the article. Further inquiries can be directed to the corresponding author.

## Abbreviations

The following abbreviations are used in this manuscript:

MASLD	Metabolic Dysfunction-Associated Steatotic Liver Disease
MASH	Metabolic Steatohepatitis
CVDs	Cardiovascular Diseases
BMI	Body Mass Index
WC	Waist Circumference
CAP	Controlled Attenuation Parameter
LSM	Liver Stiffness Measurement
PAI-1	Plasminogen Activator Inhibitor-1
FSG	Fasting Serum Glucose
LDL-C	Low-Density Lipoprotein Cholesterol
HDL-C	High-Density Lipoprotein Cholesterol
AST	Aspartate Aminotransferase
ALT	Alanine Aminotransferase
γGT	Gamma-Glutaminyl Transferase
HbA1c	Glycated Hemoglobin
HOMA-IR	Homeostasis Model Assessment of Insulin Resistance
IPAQ	International Physical Activity Questionnaire
PREDIMED	Prevention with Mediterranean Diet
FM	Fat Mass
FFM	Fat-Free Mass
TBW	Total Body Water
